# Nicotine stimulates collagen type I expression in lung via α7 nicotinic acetylcholine receptors

**DOI:** 10.1186/s12931-017-0596-8

**Published:** 2017-06-02

**Authors:** Glenn W. Vicary, Jeffrey D. Ritzenthaler, Tanmay S. Panchabhai, Edilson Torres-González, Jesse Roman

**Affiliations:** 1 Department of Pharmacology and Toxicology, University of Louisville School of Medicine, Louisville, KY USA; 2 Department of Medicine, Division of Pulmonary, Critical Care and Sleep Disorders, University of Louisville School of Medicine, 550 South Jackson Street (3rd floor), Louisville, KY USA; 3 Louisville Robley Rex Veterans Affairs Medical Center, Louisville, KY USA

**Keywords:** Tobacco, Nicotine, Lung, Matrix, Integrin, Tissue remodeling, Collagen

## Abstract

**Background:**

Tobacco-related chronic lung diseases are characterized by alterations in lung architecture leading to decreased lung function. Knowledge of the exact mechanisms involved in tobacco-induced tissue remodeling and inflammation remains incomplete. We hypothesize that nicotine stimulates the expression of extracellular matrix proteins, leading to relative changes in lung matrix composition, which may affect immune cells entering the lung after injury.

**Methods:**

Pulmonary fibroblasts from wildtype and α7 nicotinic acetylcholine receptor knockout (α7KO) mice were exposed to nicotine and examined for collagen type 1 mRNA and protein expression. Testing the potential role on immune cell function, pulmonary fibroblasts were retained in culture for 120 h. The fibroblasts were eliminated by osmotic lysis and the remaining matrix-coated dishes were washed thoroughly. U937 cells were incubated on the matrix-coated dishes for 24 h followed by evaluation of IL-1β gene expression. Wildtype or α7KO C57BL/6 mice (female, 8–12 weeks) were fed normal diet and exposed to nicotine in their drinking water (100 μg/ml) for 8-12weeks. Lungs were processed for mRNA, protein, and histology. Statistical significance was determined at *p* ≤ .05 by two-tailed test or 2-way ANOVA with Bonferroni posttest.

**Results:**

We found that nicotine stimulated collagen type I mRNA and protein expression in a dose-dependent manner and up to 72 h in primary lung fibroblasts. The stimulatory effect of nicotine was inhibited in α7KO primary lung fibroblasts. Testing the potential role of these events on immune cell function, U937 monocytic cells were cultured atop matrices derived from nicotine-treated lung fibroblasts. These cells expressed more IL-1β than those cultured atop matrices derived from untreated fibroblasts, and antibodies against the α2β1 collagen integrin receptor inhibited the effect. Nicotine also stimulated fibroblast proliferation via MEK-1/ERK, unveiling a potentially amplifying pathway. In vivo, nicotine increased collagen type I expression was detected in wildtype, but not in α7KO mice. Wildtype mice showed increased collagen staining in lung, primarily around the airways.

**Conclusions:**

These observations suggest that nicotine stimulates fibroblast proliferation and their expression of collagen type I through α7 nAChRs, thereby altering the relative composition of the lung matrix without impacting the overall lung architecture; this may influence inflammatory responses after injury.

## Background

Tobacco is considered the single greatest cause of preventable death in the world [1]. Chronic tobacco exposure is associated with the development of chronic lung disorders like chronic obstructive pulmonary disease and idiopathic pulmonary fibrosis, and is the main cause of lung cancer globally [1]. A larger effect of tobacco smoke inhalation is the induction of inflammation, a process characterized by the release of soluble mediators, oxidant stress, and the recruitment of inflammatory cells into tissue [2]. This promotes tissue remodeling, including alterations in lung structure and function, and oncogenesis [3].

Due to nicotine’s well known addictive effects, attention has turned to this plant derived alkaloid which represents ~0.6–3.0% of the dry weight of tobacco. Recent studies have unveiled effects on lung development [4, 5] and on inflammatory processes [6]. This is possible because of the existence of nicotinic receptors in lung tissue capable of signal transduction [7]. These receptors, termed nicotinic acetylcholine receptors (nAChRs), are multimeric acetylcholine-triggered channel proteins that form homomeric and heteromeric α and β chain pentameters. The α7 nAChR is the most abundant homomeric receptor form, composed of five α7 subunits. In developing primate lungs, α7 nAChRs were detected in airway epithelial cells, around large airways and blood vessels, free alveolar macrophages, alveolar type II cells, and pulmonary neuroendocrine cells [4]. Additionally, increased α7 nAChR expression has been shown with nicotine administration in bronchial epithelial and endothelial cells as well being implicated in regulation of inflammation and cancer [8–11].

Our laboratory has previously demonstrated that nicotine stimulates lung fibroblasts to express fibronectin by acting on α7 nAChRs both in vitro and in vivo [11]. Fibronectin is a matrix glycoprotein, which is highly expressed in injured tissues, and is considered a sensitive marker of tissue injury and activation of tissue remodeling [12, 13]. In an injured lung, fibronectin is deposited over denuded basement membranes where it is thought to support the migration of alveolar epithelial cells during repair [13]. The excessive deposition of fibronectin, however, has been hypothesized to promote disrepair [14]. Human studies also show increased fibronectin content in the bronchoalveolar lavage fluid of smokers [15]. However, nicotine-induced fibronectin expression and deposition are not sufficient to alter overall lung architecture and, to date, there are no data suggesting nicotine, alone, promotes significant lung tissue remodeling.

In this study, nicotine is shown to stimulate lung fibroblasts to express collagen type I. This fibrillar collagen is another extracellular matrix component highly expressed in tissues during injury and repair, and its expression signals activation of tissue remodeling. Collagen type I is the most abundant form of collagen in the human body and is present in connective tissue throughout the body including tendon, ligament, skin, and lung tissue. Each rope-like procollagen molecule is made up of three chains: two pro-α1 (I) chains, which are produced from the *COL1A1* gene, and one pro-α2 (I) chain, which are produced from the *COL1A2* gene. After processing, the resulting mature collagen molecules arrange themselves into long, thin fibrils. Individual collagen molecules are then cross-linked to one another within these fibrils thereby forming strong collagen fibrils. Studies performed in vivo confirmed nicotine induction of collagen type I without changes in overall lung architecture in lung matrix. Also, we found that nicotine-treated fibroblasts produce a collagen-containing matrix capable of stimulating monocytic cells to produce the pro-inflammatory cytokine IL-1β in vitro.

Together, these observations suggest that nicotine stimulates alterations in the relative composition of the lung extracellular matrix favoring fibronectin [11] and collagen type I (this report) expression without altering the overall tissue architecture of the lung. These subtle changes may render the host susceptible to excessive tissue damage after injury.

## Methods

### Reagents

The Mitogen-enhanced kinase-1 (MEK-1) inhibitor PD98059 was purchased from New England Biolabs, Inc. (Beverly, MA). Mouse α7 nAChR siRNA and control non-target siRNAs and Real-Time Quantitative PCR primers (QuantiTect Primer Assays) used to quantify mRNA levels by Real-Time RT-PCR were purchased from Qiagen (Valencia, CA). Polyclonal antibodies against the murine α7 nAChR, and MG 624, an α7 nAChR inhibitor, were purchased from Santa Cruz Biotechnology, Inc. (Santa Cruz, California). All other reagents were purchased from Sigma Chemical Company (St. Louis, MO) or Fisher Scientific (Pittsburgh, PA) unless otherwise specified.

### Cell culture and treatment

Primary lung fibroblasts (used between 3 and 7 passages) were harvested from wildtype control or α7 nAChR deficient C57BL/6 mice (α7KO) (Jackson Laboratories, Bay Harbor, MA) and cultured in DMEM (10% FBS) (Cellgro, Manassas, VA) as previously described [11, 16]. α7 nAChR knockout was verified by RT-PCR and Western Blot (Fig. 2a). The doses of nicotine (1–75 μg/ml) were chosen based on previous experiments and the published literature [11, 17]. Cell viability was determined by Trypan Blue exclusion.

### Silencing of nAChRs and detection of mRNAs by RT-PCR

Primary lung fibroblasts were plated onto 12-well plates (4 × 10^4^ cells/well) and incubated in DMEM (10% FBS) for 24 h. Fibroblasts were transfected with α7 nAChR or control non-target siRNA (150 ng) according to the manufacturer’s protocol using HiPerFect Transfection Reagent (Qiagen). Transfected fibroblasts were treated with 50 μg/ml nicotine for up to 72 h. RNA was extracted from cells or lung tissue using the reagent RNAzol B™ (Tel-test Inc., Friendswood, TX). Real-time PCR was performed as previously described [17] utilizing the primers to mouse collagen type I, 18S, IL-1β, and α7 nAChR in a SmartCycler™ system (Cepheid Sunnyvale, CA). Primer sequences are as follows: Mouse collagen type I forward (5’-GTGCTGTTGGTGCTGCTG), reverse (5’-CAGGAGCACCAGCAATAC); 18S forward (5’-GTGACCAGAGCGAAAGCA), reverse (5’-ACCCACGGAATCGAGAAA); IL-1β forward (5’-GAGCACCTTCTTTTCC), reverse (5’-CTGGTGGAAGAAAAGG), probe; and α7 nAChR forward (5’-CTGCTGGGAAATCCTAGGCACACTTGAG or GACAAGACCGGCTTCCATCC), reverse (5’-CCTGGTCCTGCTGTGTTAAACTGCTTC). Negative controls consisted of dH_2_O and RNA without primers. Bioluminescent RT-PCR was accomplished according to a published method [18]. Values were normalized to 18S and expressed as relative change vs. untreated mouse lung tissue.

### Protein detection via Western blotting

Western blots were performed as previously described [11, 17]. Collagen blots were run in native conditions and GAPDH in denaturing conditions. Blots were incubated with primary polyclonal antibody against either GAPDH (Abcam) (1:5000 dilution), collagen type I (ACRIS, San Diego, CA or Abcam, Cambridge, MA) (1:1000), total and p-Smad (Rockland Immunochemicals, Gilbertsville, PA) (1:2000), total and p-ERK 1&2 (Cell Signaling, Beverly, MA) (1:1000), and α7 nAChR (Sigma) (1:500). Blots were then incubated with secondary goat anti-rabbit IgG (HRP-conjugated) (Sigma/Li-CORE, Lincoln, NE) (1:20000). Fluorescent blots were read on a LI-COR Odyssey CLx imaging system.

### Protein detection via immunohistochemistry

Immunohistochemistry was performed as previously described [19]. Briefly, application of primary IL-1β antibody (Abcam, 1:100 dilution) incubated 4 °C overnight. Enzyme-conjugated secondary antibodies were applied, and the specific staining was visualized after the addition of the enzyme-specific substrate.

### Cell viability assay

Wildtype or transfected primary lung fibroblasts (1 × 10^4^ cells/ml) were added to 96-well tissue culture plates and incubated at 37 °C for up to 72 h with nicotine (50 μg/ml) in the presence or absence of PD98059 (50 μM). Afterwards, the luminescence of viable cells was detected using Cell Titer-Glo Luminescent Cell Viability Assay Kit according to the manufacturer protocol (Promega).

### Matrix deposition and IL-1β measurement

Fibroblasts were treated with 50 μg/ml of nicotine, PD98059 (50 μM), or MG 624 (10 μM) and retained in culture for 120 h. Afterwards, the fibroblasts were eliminated by osmotic lysis and the remaining matrix-coated dishes were washed thoroughly [20]. U937 cells permanently transfected with the human IL-1β gene promoter connected to a luciferase reporter gene were incubated on the matrix-coated dishes for 24 h followed by evaluation of IL-1β gene expression by luciferase as previously described [21]. Mouse IgG or anti-α2β1 integrin antibodies were cultured with U937 cells for 1 h before plating on isolated matrices (Abcam).

### Animal treatments

Wildtype or α7KO C57BL/6 mice (female, 8–12 weeks old) (Jackson Laboratories) were fed normal diet and exposed to nicotine in their drinking water (100 μg/ml) for 8-12 weeks as described by Rowell et al. [22, 23]. Mice were euthanized followed by RNA and protein isolation along with *en bloc* isolation of the lungs, which were inflated at standard pressure, fixed in formalin, paraffin-embedded, and sectioned (5 μm) for histological analysis. The appropriate institutional committees approved all animal studies.

### Histological and cytological analysis

Lung sections were stained using Weigert's iron hematoxylin for 10 min, rinsed, treated with Biebrich scarlet-acid fuchsin solution for 10 min, washed and transferred to aniline blue stain for 30–60 min (Masson’s Trichrome Staining Kit, Richard-Allan Scientific, Kalamazoo, MI); or stained with 5% Sirius Red (Polysciences Inc, Warrington, PA)/Fast Green (Achros, New Jersey) saturated with picric acid for 30 min. The Masson’s trichrome slides were blindly graded on their intensity of collagen staining by 8 investigators based on a scale of 0–3 as previously described [24].

### Statistical evaluation

All experiments were repeated at least 3 times. Standard deviations of the mean were calculated for all experimental values. Significance was assessed using *p* values <0.05 obtained by 2-way ANOVA with Bonferroni posttest or two-tailed *t*-test.

## Results

### Nicotine stimulates the expression of collagen type I

**Fig. 1:**
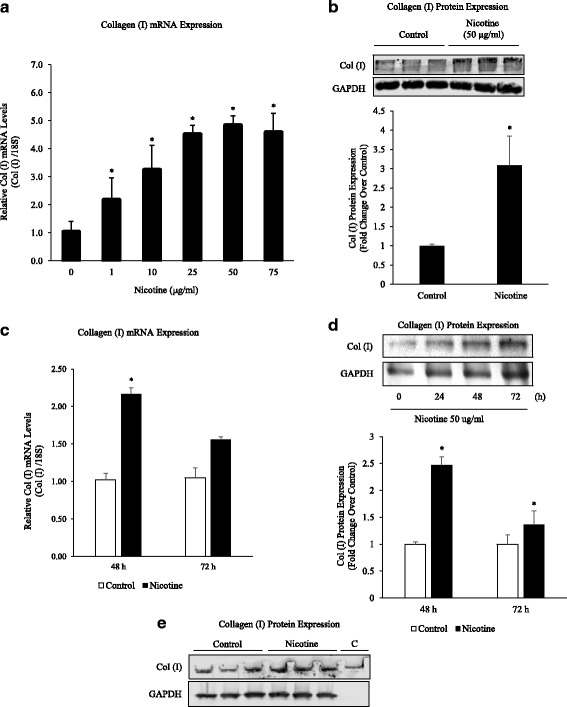
Nicotine Stimulates Collagen Type I mRNA and Protein Expression. **a** Primary lung fibroblasts (1 × 10^6^ cells/6 well) were treated with nicotine (1–75 μg/ml) for 24 h. Real-time PCR reactions were performed using mouse collagen type I or 18S primers. Note that nicotine induced a dose-dependent increase in collagen type I mRNA expression. mRNA levels were normalized to 18S and collagen type I levels compared to untreated controls. **b** Nicotine-treated fibroblasts were subjected to Western blot analysis using anti-collagen type I antibody or GAPDH, followed by secondary goat anti-rabbit IgG (IRDye®). Protein bands were separated by native (collagen type 1) or SDS-PAGE (GAPDH) gel electrophoresis (8%) and quantified by densitometry. Collagen type I levels were elevated in the presence of nicotine (50 μg/ml). **c** Primary lung fibroblasts were treated with nicotine (50 μg/ml) for up to 72 h. Nicotine induced a significant increase in collagen type I mRNA levels at 48 h. **d** Fibroblasts were treated with nicotine (50 μg/ml) for up to 72 h. Protein bands were separated by SDS-PAGE gel electrophoresis (8%) and quantified by densitometry. Collagen type I protein was increased at 48 and 72 h by Western blot analysis. **e** Purified rat-tail collagen **c** was run with 72-h protein samples for antibody validation. Quantification of 1E included in 1D. All experiments repeated at least 3 times. Significance was assessed using *p* values <0.05 obtained by two-tailed *t*-test

While exploring the effects of nicotine on lung cells, we found that nicotine stimulated the expression of collagen type I in lung fibroblasts. As depicted in Fig. 1, nicotine induced the expression of collagen type I mRNA in a dose-dependent fashion (Fig. 1a). Maximum mRNA expression was observed at 50 μg/ml of nicotine. Twenty-four hours after nicotine stimulation, increased collagen type I protein was detected in cell extracts (Fig. 1b). Nicotine also stimulated collagen type I mRNA and protein expression up to 72 h (Fig. 1c and d).

### Role of α7 nAChRs in collagen expression and cellular proliferation

**Fig. 2:**
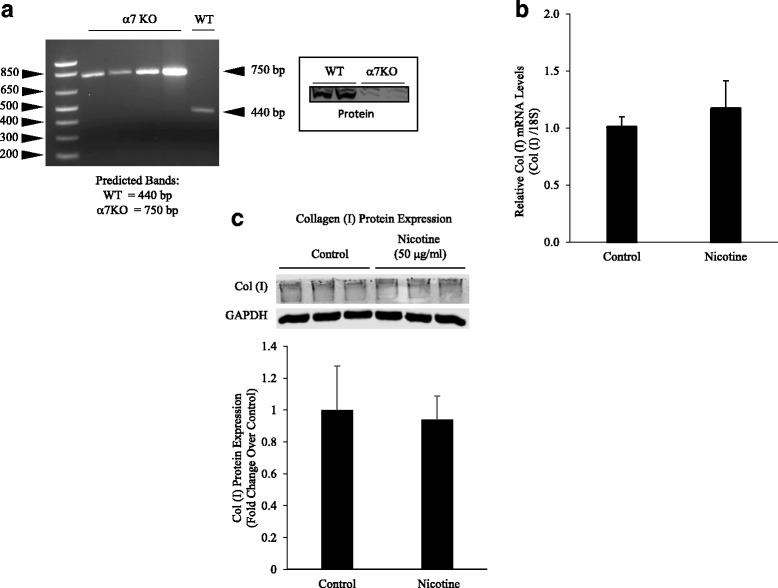
Nicotine acts through α7 nAChRs. **a** The absence of α7 nAChR was verified by mRNA and protein expression in the α7KO mice. **b** α7KO fibroblasts were exposed to nicotine for 24 h and PCR run for collagen type I mRNA expression. Nicotine failed to stimulate collagen type I mRNA expression in α7KO cells. **c** Nicotine-treated α7KO fibroblasts were subjected to Western blot analysis using anti-collagen type I antibody or GAPDH, followed by secondary goat anti-rabbit IgG (IRDye®). Protein bands were separated by native (collagen type 1) or SDS-PAGE (GAPDH) gel electrophoresis (8%) and quantified by densitometry. Nicotine did not stimulate increased collagen deposition in α7KO fibroblasts. Experiments were repeated at least 3 times. Significance was assessed using *p* values <0.05 obtained by two-tailed *t*-test

**Fig. 3:**
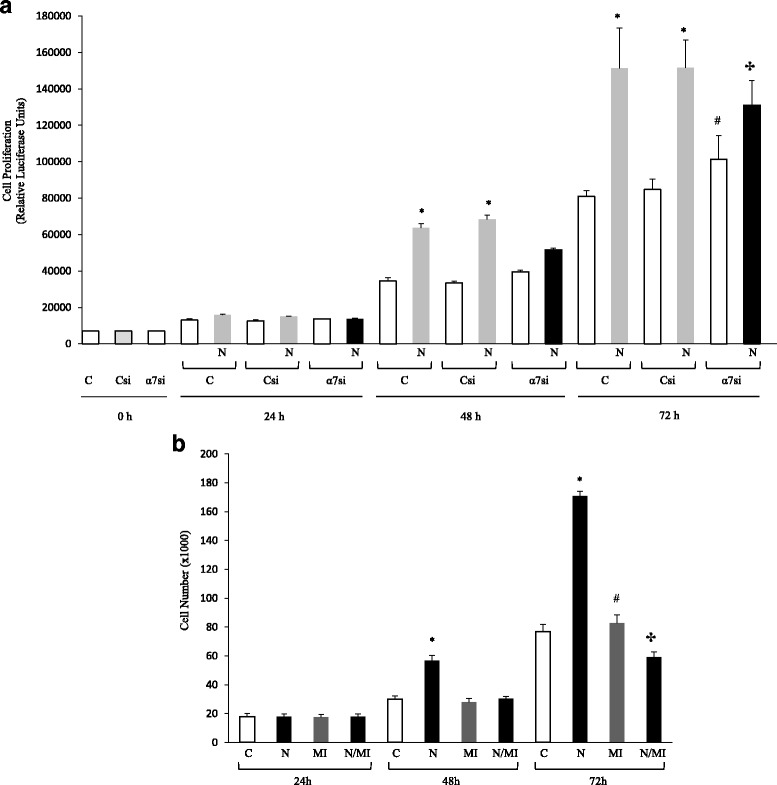
Nicotine Stimulates the Proliferation of Lung Fibroblasts via α7 nAChR-mediated Induction of ERK. **a** Lung fibroblasts were cultured for up to 72 h after transfection with control (Csi) or α7 nAChR siRNAs (α7si). Silencing of α7 nAChR was confirmed with Western blot (not shown). At the appropriate times, the experiment was halted and live cells counted. Nicotine stimulated cell proliferation at 48 and 72 h, while α7 nAChR siRNA inhibited the nicotine-induced response at 48 and 72 h when compared to nicotine-treated control cells. **b** Fibroblasts were cultured for up to 72 h with or without nicotine (50 μg/ml) in the presence or absence of PD98059 (MI, 50 μM), an inhibitor of MEK-1. Cell number was determined with the use of a Neubauer hemacytometer along with trypan blue stain. Cell viability was unchanged with treatment (Not Shown). Note that nicotine stimulated fibroblast proliferation at 48 h, but the effect was most noticeable at 72 h. The inhibitor PD98095 alone did not affect the proliferation of cells, but inhibited the stimulatory effect of nicotine. Experiments were repeated at least 3 times. Significance was assessed using *p* values <0.05 obtained by 2-way ANOVA with Bonferroni posttest

We evaluated the expression of α7 nAChR in α7KO and wildtype mice (Fig. 2a). RT-PCR verified the absence of α7 nAChRs in the α7KO mice. We then evaluated nicotine-induced collagen type I mRNA expression in primary lung fibroblasts harvested from α7KO animals. Nicotine failed to stimulate collagen type I expression in α7KO cells (Fig. 2b). Similarly, we found nicotine did not induce collagen type I protein expression in α7KO fibroblasts (Fig. 2c). Nicotine also stimulated the proliferation of lung fibroblasts and this effect was also through the α7 nAChR. As depicted in Fig. 3a, nicotine-induced proliferation was inhibited at 48 and 72 h by siRNA silencing of α7 nAChRs. Additionally, the mitogenic effects of nicotine were inhibited by PD98059, a MEK-1/ERK inhibitor (Fig. 3b).

### Nicotine increases collagen expression in vivo

**Fig. 4:**
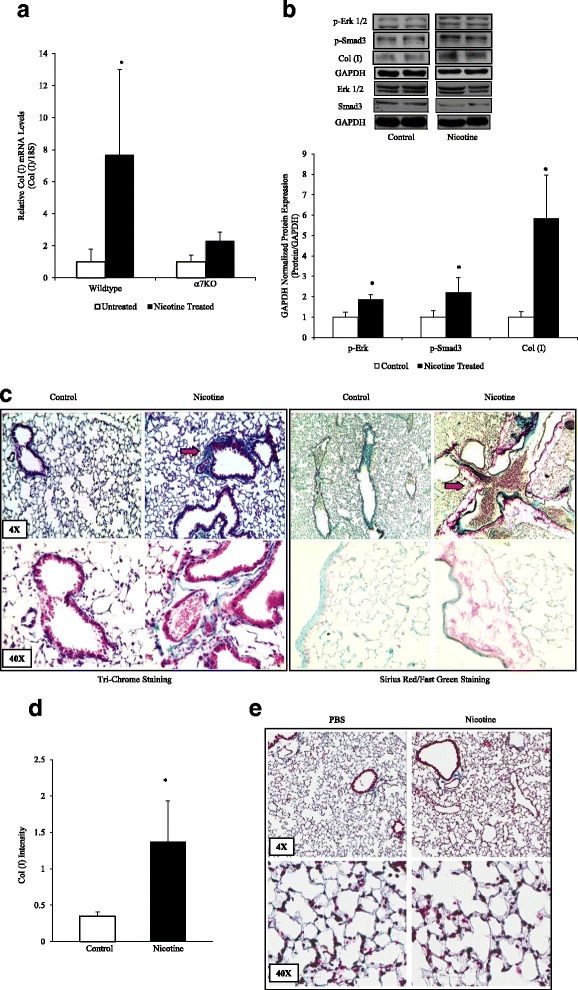
Nicotine Stimulates Collagen Expression in Lung in vivo. **a** The lungs of mice exposed to nicotine (100 μg/ml in the drinking water for 8 weeks) were isolated for RNA analysis, which showed an increase in collagen type I expression in nicotine-treated wildtype mice, but not in α7KO mice. **b** Protein analysis showed increased p-ERK 1/2, p-Smad3, and collagen type I protein expression when compared to control lungs. P-Erk, p-Smad3, and collagen type 1 were all completed on the same gel, with the blot stripped between probings. The total Erk, Smad3 gels were run on a separate gel and stripped between probings. **c** Lungs were also inflated at standard pressure, fixed in formalin, paraffin-embedded, and sectioned (5 μm) for histological analysis. Lung sections were stained using Weigert's iron hematoxylin for 10 min., rinsed, treated with Biebrich scarlet-acid fuchsin solution for 10 min., washed and transferred to aniline blue stain for 30–60 min or stained with Sirius Red/Fast Green for 30 min. Arrows indicate increased collagen type I deposition (*blue stain or Red stain*) in wildtype animals exposed to nicotine. **d** The tri-chrome slides were blindly graded on a scale of 0–3 based on the intensity of collagen staining. **e** Nicotine treatment resulted in no differences in tri-chrome staining in α7KO mice. Experiments were repeated at least 3 times. Significance was assessed using *p* values <0.05 obtained by two-tailed *t*-test

We turned our attention to studies in vivo. For this, wildtype and α7KO mice were exposed to nicotine in their drinking water (100 μg/ml) for 12 weeks to test if the mechanisms described above are relevant to the situation in vivo. This model has been shown to increase nicotine levels in blood and tissue similar to those of heavy smokers without affecting body weight [23]. Lower concentrations (<1 μg/ml) of nicotine can be found in the blood of smokers, while higher concentrations (1–10 μg/ml) are deposited in body tissues [22]. As depicted in Fig. 4a, the lungs of mice exposed to nicotine showed increased collagen type I mRNA expression. α7KO mice showed an insignificant increase in collagen type I mRNA. Protein analysis showed an increase in collagen type I, p-ERK 1/2, and p-Smad3 protein expression when compared to control lungs (Fig. 4b). Total TGF-β levels were unchanged with nicotine treatment (not shown).

Histological analysis was performed on lung tissue harvested from untreated and nicotine-treated animals and found increased collagen deposition in wildtype mice treated with nicotine, noticeably around airway and vascular structures (red arrows) in tissues submitted to both Masson’s tri-chrome and Sirius red staining (Fig. 4c). The increase in collagen was confirmed by blinded scoring of unlabeled tissue slides by eight investigators (Fig. 4d). Nicotine also failed to stimulate collagen deposition in the α7KO mice (Fig. 4e).

### Extracellular matrices derived from nicotine-treated lung fibroblasts stimulate monocytic cell expression of IL-1β

**Fig. 5:**
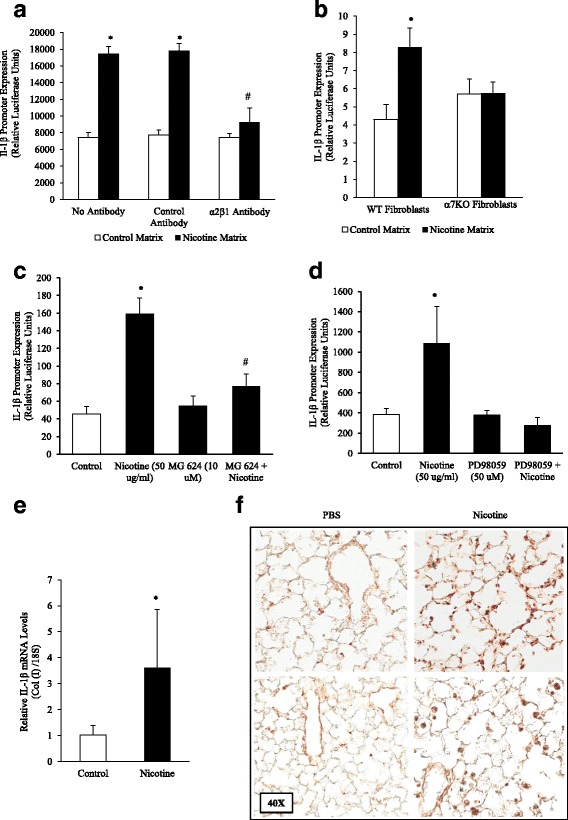
Matrices Derived from Nicotine-treated Fibroblasts and Mice Stimulate IL-1β Expression in Monocytic Cells. **a** Lung fibroblasts (5x10^4^ cells/12 well) were treated with nicotine (50 μg/ml) for 120 h. Fibroblasts were removed by osmotic lysis, the plates were washed thoroughly, and human monocytic U937 cells expressing the human interleukin-1β gene promoter connected to a luciferase reporter gene were overlaid atop the fibroblast-derived matrix. Afterwards, expression of the IL-1β promoter was analyzed by luciferase assay. We found that collagen-containing matrices derived from nicotine-treated fibroblasts stimulated monocytic cells to express the pro-inflammatory cytokine IL-1β. Furthermore, nicotine-treated fibroblast matrix induction of IL-1β was inhibited by anti-α2β1 integrin antibodies. **b** IL-1β gene transcription was not increased in U937 cells cultured on matrices derived from nicotine-treated α7 nAChR deficient primary lung fibroblast matrix over control. **c** Fibroblasts pretreated with MG 624, an α7 nAChR antagonist (10 μM), concurrently with nicotine inhibited IL-1β expression without affecting baseline expression. **d** The nicotine-treated fibroblast matrix IL-1β induction was inhibited by MEK-1 inhibitor PD98059 (50 μM), with PD98059 alone bringing IL-1β expression below baseline. **e** The lungs of mice exposed to nicotine (100 μg/ml in the drinking water for 8 weeks) were isolated for RNA analysis, which showed an increase in IL-1β gene transcription by RT-PCR. **f** Lungs from control and nicotine-treated mice were stained by immunohistochemistry for IL-1β. Increased staining was present in mice treated with nicotine. Experiments were repeated at least 3 times. Significance was assessed using *p* values <0.05 obtained by 2-way ANOVA with Bonferroni posttest

We then investigated whether matrices derived from nicotine-treated fibroblasts exert a differential effect on immune cells. For this, we cultured U937 human monocytic cells atop of matrices derived from untreated or nicotine-treated fibroblasts; U937 cells expressed the human IL-1β gene promoter fused to a luciferase reporter gene [21]. As presented in Fig. 5a, we found that IL-1β gene transcription was increased about 2-fold when cultured atop of matrices derived from nicotine-treated fibroblasts when compared to cells cultured atop matrices derived from untreated fibroblasts. This effect appeared to be mediated by collagen binding to α2β1, a collagen integrin receptor, since anti-α2β1 antibodies inhibited the induction of IL-1β (Fig. 5a). Importantly, IL-1β gene transcription was not increased in U937 cells cultured atop matrices derived from nicotine-treated α7KO lung fibroblast (Fig. 5b). Treatment of fibroblasts with MEK-1 inhibitor PD98059 or an α7 nAChR inhibitor MG 624 prior to exposure to nicotine blunted U937 IL-1β induction by the matrix (Fig. 5c and d). Lastly, this process might be relevant in vivo since whole lung RNA from nicotine-treated mice showed elevated IL-1β gene transcription and staining by immunohistochemistry (Fig. 5e and f).

## Discussion

Tobacco-related lung disease is an important health problem worldwide. Several studies suggest that tobacco exposure promotes lung tissue remodeling through oxidant stress, inflammation, and the induction of matrix-degrading proteases, among other mechanisms. The latter is supported by animal studies showing that overexpression of matrix metalloproteinase (MMP)-1 promotes the development of emphysema in transgenic mice, while the lack of MMP-12 is protective [25, 26]. Furthermore, alterations in MMPs and other proteases have been detected in humans with tobacco-related lung disease [27]. Unfortunately, this information has not yet translated into the development of effective therapeutic strategies. Here, we explore another mechanism of action, the induction of lung tissue remodeling through stimulation of extracellular matrix deposition. We hypothesized that nicotine, a major component of tobacco, is not only involved in tobacco addiction, but stimulates lung fibroblasts to release matrix components that affect the relative composition of the lung matrix. Consistent with this idea, we found that nicotine stimulates lung fibroblast expression and release of collagen type I up to 72 h.

Previously, we reported that nicotine stimulates the expression in lung of fibronectin, a matrix glycoprotein implicated in injury and repair [11]. However, fibronectin matrices are often considered ‘*transitional*’ matrices whose deposition does not necessarily lead to irreversible changes in tissue architecture in the absence of other factors. The discovery that nicotine also stimulates the deposition of fibrillar collagens (this report) is important because it suggests that the effects of nicotine on matrix composition may be more permanent. Collagen type I is highly expressed in injured lungs as demonstrated in acute lung injury, COPD, and chronic fibrotic lung disorders [28]. Additionally, collagen has been associated with extralobar pulmonary artery stiffening caused by chronic hypoxia [29], induction of epithelial-to-mesenchymal transition in non-small cell lung cancer cell lines [30], and stimulation of cell chemotaxis by its fragments [31].

Considering the importance of the proposed mechanisms of action, we turned our attention to the pathways involved in stimulation of collagen expression. We found that nicotine affected collagen expression in lung fibroblasts by acting on α7 nAChRs. Several studies have implicated α7 nAChRs in lung branching morphogenesis and in the pathogenesis of lung cancer [32, 33]. More recently, α7 nAChRs were found to mediate the effects of nicotine in developing lungs [5, 34]. Specifically, morphological airway abnormalities and airflow limitation were detected in the offspring of nicotine-treated wildtype animals, but not in animals lacking α7 nAChRs. Interestingly, and reminiscent to our work, collagen was found to be upregulated around the airways of animals exposed to nicotine. Based on the information presented here and the growing number of publications implicating α7 nAChRs in several disease states, it is reasonable to consider α7 nAChRs as promising targets for drug development to counteract the deleterious effects of tobacco. This is now possible considering that the technology to develop safe and effective agents that target nAChRs are currently available for human use [35].

Another important finding was that nicotine also stimulated fibroblast proliferation, a process capable of further promoting tissue remodeling. This effect was also mediated via α7 nAChRs as demonstrated by the lack of response in cells silenced for α7 nAChRs. In prior work, we and others demonstrated that nicotine leads to ERK activation [11]. Consequently, we tested the role of ERK and found that a MEK-1/ERK inhibitor, PD98059, inhibited nicotine-induced fibroblast proliferation.

To determine the potential relevance of our findings, we tested nicotine exposure in vivo. Previous studies have shown nicotine increases collagen type I expression in vivo in rhesus monkeys [36]. We exposed mice to nicotine in their drinking water for 8-12 weeks. When examined, the harvested lungs from nicotine-treated wildtype mice showed increased collagen deposition predominating around airway and vascular structures as determined by immunohistochemistry. Consistent with our in vitro findings, lung tissue also showed increased phosphorylation of ERK. We also detected increased phosphorylation of Smad3, a transcription factor known for mediating many of the pro-fibrotic effects of transforming growth factor-β. Increased Smad3 has been associated with collagen expression in cardiac and dermal fibroblasts [37, 38].

Our findings suggest that nicotine can promote fibronectin [11] and collagen type I (this report) deposition in lung without affecting the organ’s overall architecture. We refer to this process, which appears to be self-limiting, as “transitional remodeling”. Since deposition of new collagen fibrils in our model was not associated with dramatic alterations in lung architecture, how then do these subtle changes in lung matrix composition affect the lung? We reasoned that newly deposited collagen fibrils do not affect the lung in the absence of other injurious stimuli, but instead, may influence immune cell function after injury. Consistent with this idea, our lab has previously shown that purified collagen type I can robustly activate monocytic IL-1β expression [39]. In this report, we show that collagen-containing matrices derived from nicotine-treated fibroblasts are capable of activating monocytic cells and stimulating their expression of the pro-inflammatory cytokine IL-1β. This pro-inflammatory event was inhibited by pretreatment of fibroblasts with antibodies against α2β1, a collagen-binding integrin [40], PD98059 (MEK-1 inhibitor), MG 624 (α7 nAChR inhibitor), or matrices derived from nicotine-treated α7KO primary lung fibroblasts when compared to control. In vivo, increased IL-1β gene expression and staining was also detected in the lungs of nicotine-treated mice (Fig. 5e and f). The increased IL-1β staining detected in nicotine-treated lungs is likely on macrophages, which nicotine has previously been shown to activate [41]. This suggests that nicotine has the capacity to activate resident macrophages in addition to recruiting circulating macrophages. Integrins also control immune responses in T cells. For example, integrin-mediated binding to collagen provides a co-stimulatory signal for T cell activation [42], resulting in increased proliferation and secretion of pro-inflammatory cytokines such as TNF-α and IFN-γ [43]. Thus, by promoting subtle alterations in matrix composition, nicotine may indirectly stimulate the exaggerated expression of pro-inflammatory cytokines (e.g., IL-1β) by immune cells recruited to the lung after injury, thereby helping perpetuate inflammation, a process considered important in the pathogenesis of tobacco-related lung disorders.

Elements of lung transitional remodeling have also been demonstrated in alcohol-exposed rats and mice [17, 44], alcoholic subjects [45], post-lung transplant recipients [46], and aging mice [14]. However, the implications of lung transitional remodeling are unknown. It is presumed that if the stimulating agent is eliminated, a ‘normal’ matrix is restored. In contrast, persistence of the transitional matrix may lead to ineffective repair after injury through the induction of pro-inflammatory agents directly or via the release of matrix fragments [47]. We and others have suggested that these changes may explain the increased incidence and mortality observed for acute lung injury in alcoholics [20, 45], the predisposition to lung cancer in smokers [8, 48], the development of rejection after lung transplantation [46], and the worse outcomes observed in elderly patients with pulmonary disorders [14].

Additionally, it is important that we emphasize the implications of this research to understanding the potential impact (and safety) of e-cigarettes, an area that remains relatively unexplored. Consistent with this report, early studies suggested that e-cigarettes cause similar cell changes as those caused by tobacco exposure [49]. Considering this, in addition to serious concerns about their ability to serve as a gateway drug for smoking and current non-smokers, the use of these agents might pose a serious setback for global health with the use of e-cigarettes in adolescents, which has doubled yearly [50, 51].

However, until further studies are performed, these statements remain highly speculative. Booth et al., recently published a technique to isolate acellular lungs, which could provide the methods to study this interaction in a ‘*transitional matrix*’ lung [52]. Nevertheless, the idea that lung transitional remodeling may precede processes such as COPD, acute lung injury and pulmonary fibrosis, among other disorders, is tantalizing and testable.

## Conclusion

In summary, chronic nicotine exposure in mice results in lung matrix remodeling characterized by increased collagen type I expression/deposition in lung as well as phosphorylation of ERKs and Smad3. In the absence of injury, this subtle change in matrix composition does not affect the overall lung architecture, but may promote exaggerated inflammatory (e.g., induction of IL-1β by immune cells) and repair (e.g., fibroproliferation) responses after injury. These events appear to be mediated via α7 nAChRs, which may represent promising targets for intervention if lung transitional remodeling is proven to be a pre-disease susceptibility state that precedes (and promotes) lung destruction after injury.

## Abbreviations

COPD: Chronic obstructive pulmonary disease
Erk: Extracellular signal-regulated kinases
IFN-g: Interferon gamma
IL-1β: Interleukin-1β
KO: Knockout
MMP: Matrix metalloproteinase
mRNA: Messenger ribonucleic acid
nAChR: Nicotinic acetylcholine receptors
TNFα: Tumor necrosis factor-alpha
